# Recognition of motor intentions from EEGs of the same upper limb by signal traceability and Riemannian geometry features

**DOI:** 10.3389/fnins.2023.1270785

**Published:** 2023-10-30

**Authors:** Meng Zhang, Jinfeng Huang, Shoudong Ni

**Affiliations:** ^1^School of Mechanical and Power Engineering, Nanjing Tech University Nanjing, Nanjing, China; ^2^Faculty of Human Sciences, University of Tsukuba, Ibaraki, Japan; ^3^Research Institute, NeuralEcho Technology Co., Ltd., Beijing, China

**Keywords:** EEG source localization, motor imagery, Riemannian geometry, same upper limb, temporal variability

## Abstract

**Introduction:**

The electroencephalographic (EEG) based on the motor imagery task is derived from the physiological electrical signal caused by the autonomous activity of the brain. Its weak potential difference changes make it easy to be overwhelmed by noise, and the EEG acquisition method has a natural limitation of low spatial resolution. These have brought significant obstacles to high-precision recognition, especially the recognition of the motion intention of the same upper limb.

**Methods:**

This research proposes a method that combines signal traceability and Riemannian geometric features to identify six motor intentions of the same upper limb, including grasping/holding of the palm, flexion/extension of the elbow, and abduction/adduction of the shoulder. First, the EEG data of electrodes irrelevant to the task were screened out by low-resolution brain electromagnetic tomography. Subsequently, tangential spatial features are extracted by the Riemannian geometry framework in the covariance matrix estimated from the reconstructed EEG signals. The learned Riemannian geometric features are used for pattern recognition by a support vector machine with a linear kernel function.

**Results:**

The average accuracy of the six classifications on the data set of 15 participants is 22.47%, the accuracy is 19.34% without signal traceability, the accuracy is 18.07% when the features are the filter bank common spatial pattern (FBCSP), and the accuracy is 16.7% without signal traceability and characterized by FBCSP.

**Discussion:**

The results show that the proposed method can significantly improve the accuracy of intent recognition. In addressing the issue of temporal variability in EEG data for active Brain-Machine Interfaces, our method achieved an average standard deviation of 2.98 through model transfer on different days’ data.

## 1. Introduction

Due to diseases, accidents, aging, and other factors, there is an increasing proportion of the population with sensory impairments, perceptual disorders, physical disabilities, and brain degeneration, which leads to a decrease in self-care ability (Kitamura et al., 2021). This puts significant economic pressure on families and society and poses challenges to healthcare and social security systems (Norrbom and Stahl, 2022). Brain-machine interface (BMI) technology based on motor imagery (MI) provides a new solution for individuals with motor and communication disabilities (Remsik et al., 2016; Lazarou et al., 2018; Tortora et al., 2020). Compared to interaction modalities such as sound and touch, non-invasive BMI offers a more direct and natural way of interaction (Pfurtscheller and Neuper, 2001; Schalk et al., 2004). It utilizes motor imagery-electroencephalographic (MI-EEG) signals, which are brain signals generated by subjective motor intentions (Pfurtscheller and Neuper, 1997). MI-based BMI devices, such as neural prostheses and arm exoskeletons, can help individuals restore normal functionality (King et al., 2013; Cheah et al., 2021). However, this BMI task is typically limited to four classes corresponding to different body parts, such as the left and right hands, both legs, and the tongue. There has been limited research on detecting different motor tasks of the same limb, especially movements of the same joint, which restricts the freedom of MI tasks.

Some potential limitations of EEG may be contributing factors to the lag in this type of research. Firstly, EEG has a lower spatial resolution, with sensor spacing typically around 10 millimeters (Hochberg and Donoghue, 2006). Each sensor records the activity of thousands of neurons or more, which are filtered and superimposed. This limited spatial resolution poses challenges in decoding single-joint movements from a single limb using EEG since it triggers tightly packed cortical motor areas. Secondly, the number of motor cortex neurons involved in single-joint movements is fewer compared to those involved in large limb movements, and the muscle innervation of the ipsilateral upper limb originates from the brachial plexus, which largely overlaps in the cortical areas. The spatial resolution of EEG cannot reach the level of invasive surgery (Nunez and Srinivasan, 2006). These facts indicate that the signal-to-noise ratio and bandwidth of EEG signals are limited, making it challenging to decode fine-grained MI tasks.

Currently, the mainstream algorithm for MI classification tasks is Common Spatial Patterns (CSP) (Ramoser et al., 2000). Wu et al. (2013) used CSP to extract features from EEG and applied Linear Discriminant Analysis (LDA) for MI classification, achieving an average classification accuracy of 80% on two datasets. Several algorithms derived from CSP have also been proposed, such as Filter Bank CSP (FBCSP) (Ang et al., 2012), Sub-Band CSP (Zhang et al., 2015), and Common Spatio-Spectral Patterns (CSSSPs) (Reddy et al., 2019). Additionally, research has been conducted to enhance the accuracy of MI classification tasks by improving the signal-to-noise ratio. For example, Qin et al. proposed the use of Independent Component Analysis (ICA) to extract signal components related to left or right MI tasks. They reconstructed the equivalent neural sources of MI using source analysis methods and classified them based on inverse solutions, achieving an accuracy of 80% (Qin et al., 2004). Traditional EEG feature extraction algorithms have also been widely applied to MI tasks, including power spectral analysis (Lee et al., 2019), autoregressive coefficients (Talukdar et al., 2014), wavelet decomposition (Cheng et al., 2019), and Empirical Mode Decomposition (EMD) (Wang and He, 2004; Rafik and Larbi, 2019).

There has been relatively limited research on the challenging task of same upper limb MI. Xu et al. proposed the use of phase synchronization information to classify MI EEG signals of different joint actions (grasping, elbow flexion/extension, and forearm protraction) within the same limb, achieving a classification accuracy of 42.7% for the three joint actions (Xu et al., 2019). Yong et al. utilized three feature extraction algorithms (CSP, FBCSP, logarithmic band power) to decode the resting state and two MI tasks (grasping and elbow movement) of the same limb in data from 12 participants, achieving an average accuracy of 60.7% (Yong and Menon, 2015). The same team further proposed the use of autoregressive model coefficients, waveform length, and root mean square as time-domain features, combined with a Support Vector Machine classification algorithm, which improved the accuracy by 16.2% (Tavakolan et al., 2017). Ofner et al. (2017) conducted a study on low-frequency EEG time-domain features using data from 15 participants to decode six types of same-side upper limb actions for both execution and MI tasks, the classification accuracy for these six action classes was 27%. From these studies, it is evident that as the difficulty and number of MI tasks increase, the classification accuracy decreases significantly.

Recently, Riemannian geometry methods, as a novel feature representation learning tool, have been successfully applied in the field of BMI based on EEG (Yger et al., 2016). These methods have demonstrated superiority in various applications, such as sleep/respiration state classification (Navarro-Sune et al., 2016) and EEG pattern decoding (Kalunga et al., 2016). However, there has been little research on same-side upper limb MI, particularly tasks involving antagonistic movements. In the realm of motor imagery tasks, most studies have focused on left–right hand movements or coarse-grained motor imagery tasks (Shuqfa et al., 2023). These investigations have validated the applicability of active BMIs (Barachant et al., 2010a; Kumar et al., 2019), expanded research within the manifold space (Rodrigues et al., 2018), and examined the connections between spatial patterns in BMIs (Barachant et al., 2010b). However, there has been relatively little research into fine-grained tasks. While Chu, Y. et al. achieved commendable results in this regard (Chu et al., 2020), applying these findings to real-world scenarios becomes challenging due to issues such as data augmentation through dataset partitioning. Most of the applications of Riemannian geometry methods in BCIs have not critically examined the construction of the space, the substantial sensor domain often contains a plethora of noise. Riemannian geometry, a method heavily reliant on spatial configuration, imposes strict limitations on the scope of the sensor domain. Additionally, no prior investigations have explored the issue of temporal variability (Brismar, 2007) in EEG during motor imagery.

**Figure 1:**
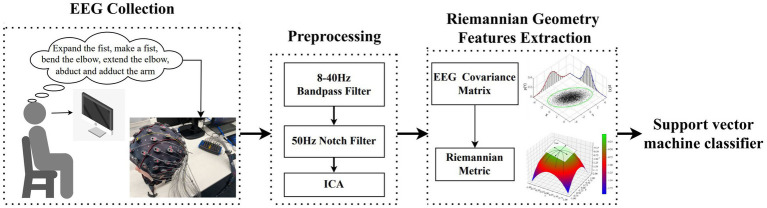
The block diagram of the proposed approach for decoding multiclass MI EEG.

The objective of this study is to increase the control degrees of freedom of non-invasive MI-BMI systems by decoding single-arm movements using EEG. The block diagram of the proposed decoding pipeline is shown in Figure 1. Independent component analysis and dipole source localization are employed to analyze EEG data during motor imagery, revealing the spatial structure of EEG correlates of movement. Feature extraction based on the Riemannian geometry of the EEG manifold successfully decodes single-arm movements and improves classification accuracy compared to other methods. This contributes to the development of non-invasive MI-BMIs with enhanced control and complex motor functionality. We also conducted in-depth investigations into the temporal variability of EEG.

## 2. Materials and methods

### 2.1. Experimental protocol and data acquisition

Fifteen healthy male participants with ages ranging from 20 to 35 years, all with a background in neuroscience, were included in the study. All participants had normal or corrected-to-normal vision and were right-handed according to self-report. In the week leading up to the experiment, none of the participants took any neurological medications, and they had no history of neurological or significant medical conditions. Before the experiment commenced, all participants provided informed consent voluntarily and received compensation after the experiment. The study was approved by the local ethics committee of Nanjing Tech University, China.

Before the experiment, participants were asked to undergo a 1-min state adjustment period. They sat on a chair, relaxed their minds, faced the display screen at approximately 75 cm distance, and placed their hands naturally on the table. During the experiment, all cognitive tasks were performed using the non-dominant hand. The experimenter sat in front of the participant, with their screen displayed separately, constantly monitoring for any program crashes or electrode impedance stability in the participant’s screen. Participants were not allowed to produce actual motor execution during the experiment and were instructed to avoid blinking or swallowing as much as possible.

**Figure 2:**
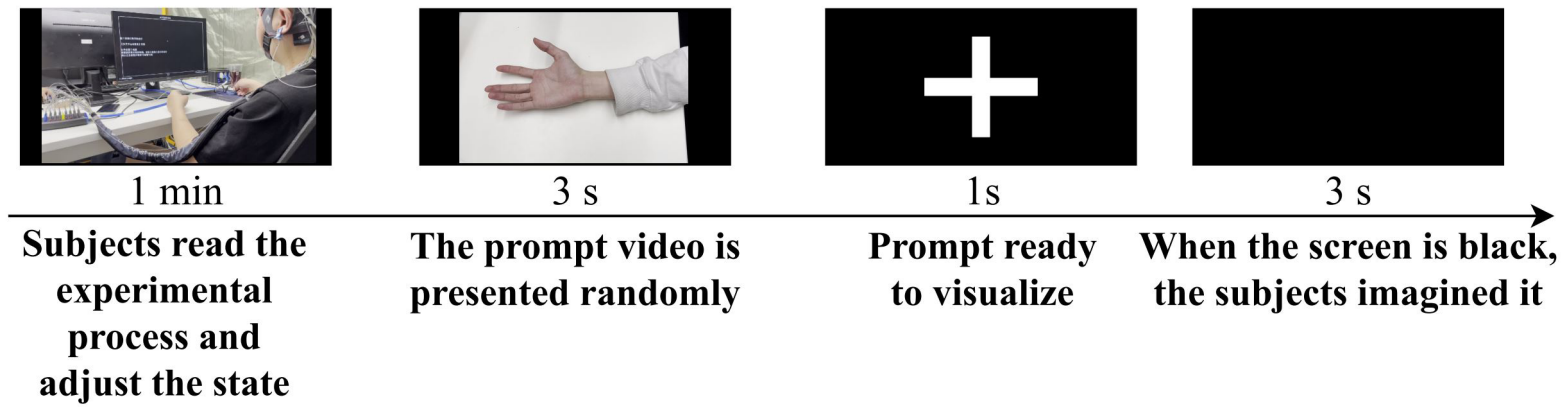
Description and sequence of events in the experimental process.

The display screen remained blank, and at the beginning of each trial, participants fixated their gaze on the screen. A 3-s instructional video was presented, and participants were required to remember the MI task shown in the video. There were six different MI tasks, including opening the hand, making a fist, flexing the elbow, extending the elbow, abducting the arm, and adducting the arm. After a “+” symbol appeared in the center of the screen, participants began to perform the same MI task as shown in the instructional video. The screen remained blank during the 3-s MI period, during which participants imagined a complete action once. This process was repeated until all categories were played, with the order of instructional videos presented randomly. Participants were allowed to take breaks between sets of experiments. Each day, ten sets of experiments were conducted, over a total of six days. Conducting the data collection experiment over six days helps prevent participants from becoming excessively fatigued during the study, thus ensuring data quality to a certain extent. Due to the temporal variability present in EEG data, it is crucial to explore the robustness of our method across different days by collecting data on separate days. The experimental procedure is illustrated in Figure 2.

The entire experiment was conducted in an electromagnetically shielded room, with experimental control carried out in a separate control room. EEG data were acquired using the g.HIAMP amplifier and g.SCARABEO wet electrodes set from g.tec company. A total of 64 electrodes were used, and their impedances were maintained below 5kΩ. Among these, 62 EEG electrodes were placed on the scalp according to the standard 10–20 system, and two reference electrodes (A1, A2) were positioned on the earlobes. The recording settings were as follows: signal sampling rate was set at 1200 Hz, high-pass filtering above 0.1 Hz was applied to remove slight interferences such as body movements, and notch filtering from 48 Hz to 52 Hz was used to eliminate power-line interference. The data acquisition was performed using both the Psychtoolbox (version: 3.0.14) and g.RECORDER software, which enabled event labeling and timestamp recording during the entire data collection. The MI-EEG dataset for each participant had a size of 360 × 64 × 3,600, representing trials, channels, and sampling points, respectively.

### 2.2. Preprocessing

The EEG data were subjected to bandpass filtering in the range of 8–30 Hz (McFarland et al., 2000) using the finite impulse response (FIR) filters from the scipy library (Neuvo et al., 1984). Both forward and backward filtering were employed to avoid phase distortion. Powerline noise at 50 Hz was removed using a notch filter with a transition band of 1 Hz (Urigüen and Garcia-Zapirain, 2015). Independent Component Analysis (ICA) based on the Infomax algorithm (Bell and Sejnowski, 1995), as implemented in the EEGLAB toolbox (Delorme and Makeig, 2004), was utilized to decompose the EEG data into independent components (ICs), ICs are set to 64. The ADJUST toolbox (Mognon et al., 2011) was employed to detect and reject ICs related to common artifacts, such as general discontinuities, electrooculogram (EOG), electromyogram (EMG) (Fatourechi et al., 2007), and electrocardiogram (ECG) (Berkaya et al., 2018), the filter threshold is set to 0.8.

### 2.3. Region of interest selection

Since the location of the signals of interest highly indicates the brain’s state, emphasizing the spatial properties during the feature extraction process is essential (Penfield and Boldrey, 1937; Friston, 2011). However, directly analyzing the spatial characteristics of EEG signals is problematic due to the volume conduction effect (Nunez and Srinivasan, 2006), which affects the collection of EEG signals. When neuronal electric fields propagate from the gray matter to the skin surface, they become distorted and diffused. Electrode coherence caused by volume conduction can be significantly mitigated through EEG source localization (source space reconstruction). Primary cortical current density can be estimated from the surface of the head to achieve source localization within a given EEG recording.

For the forward head model that couples surface voltages with internal head currents, solving the inverse problem allows obtaining the current distribution (Hallez et al., 2007). However, at each time point of EEG measurement, the distributed activity of billions of brain neurons induces scalp potential maps (Teplan, 2002; Xia and Hu, 2019). To accurately represent complex cortical activity with anatomically realistic geometries, it is necessary to use a dense source grid, often consisting of thousands of dipoles spanning the brain volume. Nevertheless, an infinite number of combinations of active neural sources can produce the same scalp surface potential maps. Due to the complexity and real-time constraints of the signal processing sequence, the inverse operation is generally approximated as a linear problem (Eom, 2023): Y = AX.

In this study, Y represents the EEG matrix after the ICA reconstruction of all participants, A is the transfer matrix obtained through g.tec’s head model, and X is the source component to be spatially localized. The method of using Low resolution electrical tomography (LORETA) (Pascual-Marqui et al., 2002) based on least squares is adopted to solve this inverse problem more accurately.


$${\hat{D}}_{\mathrm{LORETA}}=\arg\min_{D}∥M-LD{∥}_{2}+\lambda ∥BWD{∥}_{2}$$

After obtaining the source components, we calculate their power distribution topology and compute the Euclidean distance between the coordinates of clustered dipole sources and the coordinates of electrodes in the head model. We retain the EEG channels within the sensor domain that are close to the active neurons. This process allows us to select brain regions that exhibit the highest time-frequency correlation with the behavior related to the MI task.

### 2.4. Feature extraction

The matrix $X_{i}=\left[x_{t+T_{i}}\ldots x_{t+T_{i}+T_{s}-1}\right]\in R^{n\times T_{s}}$ represents the processed format of the EEG signal data, corresponding to the i-th MI experiment starting at time $t=T_{i}$ , with n denoting the retained number of electrodes. $T_{s}$ represents the number of sampling time points in each trial.

The data is transformed to the space of Symmetric Positive Definite (SPD) matrices, denoted as $P_{n}$ , using the Oracle Approximate Shrinkage Estimator to estimate the sample covariance matrix. For the i-th trial, the computation is as follows:


$$P_{i}=\frac{1}{T_{s}-1}X_{i}X_{i}^{T}$$

**Figure 3:**
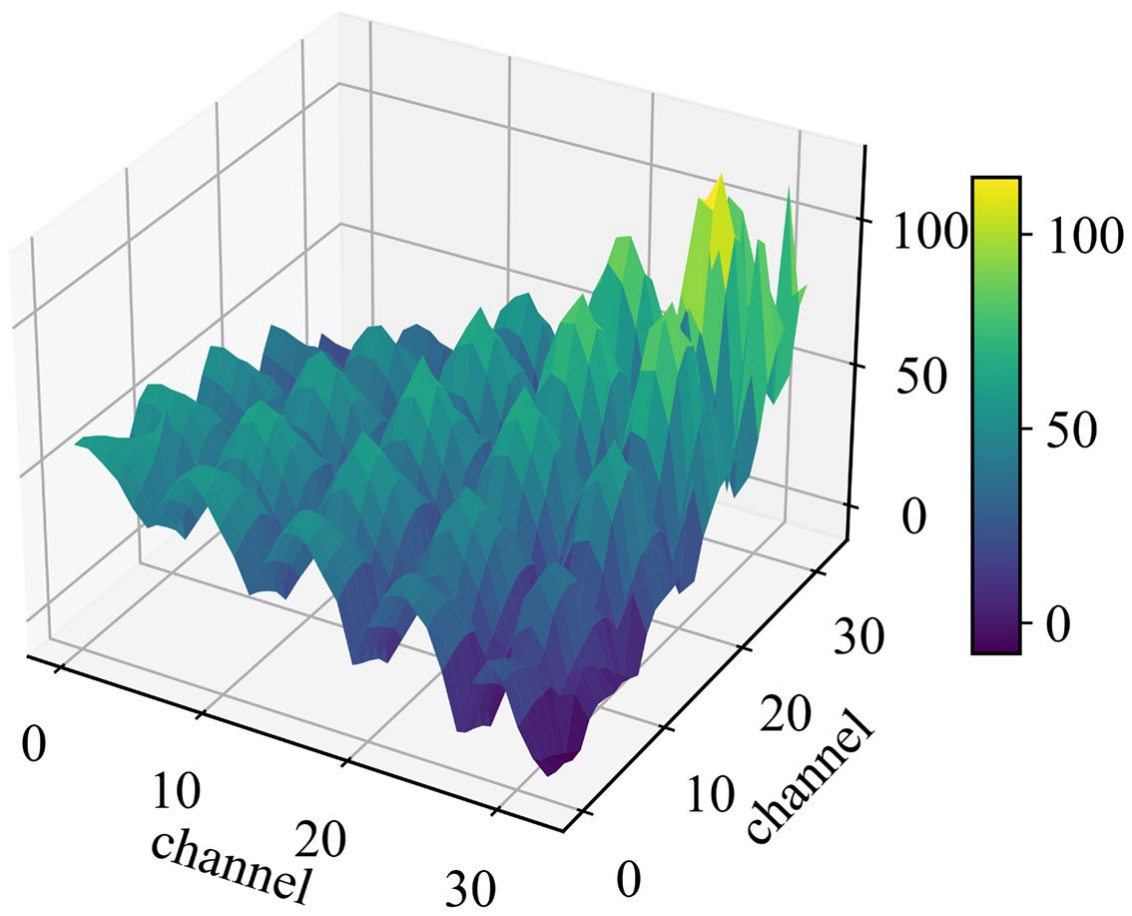
The 3D surface map of a covariance matrix. The number of the channel is the sensing domain after traceability processing.

Since the SPD matrix space is a smooth and curved space, it can be considered as a manifold, as shown in Figure 3. At each point on the manifold, the derivatives form a tangent space T, and each tangent space has an inner product. In this context, the local inner product is defined using the Riemannian metric instead of the Euclidean metric, and it is given as follows:


$$\forall A,B\in T_{X}P_{n}\;A,B_{P}=Tr\left(P^{-1}AP^{-1}B\right)$$

Where $Tr\left(\text{.}\right)$ denotes the calculation of the matrix trace. The collection $P_{n}$ of these SPD matrices forms a differentiable Riemannian manifold. On this manifold, the length of the shortest curve connecting two covariance matrices is known as a geodesic. The Riemannian metric allows us to derive the geodesic distance $\delta_{R}$ from P_1_ to P_2_, known as the Riemannian distance:


$$\delta_{R}\left(P_{1},P_{2}\right)=||\log\left(P_{1}^{-1}P_{2}\right)||{}_{F}={\left[\overset{n}{\underset{i=1}{\sum }}\;\log^{2}\lambda_{i}\right]}^{1/2}$$

According to the definition of the Riemannian distance, we can use the inverse operation to map any symmetric matrix A belonging to the tangent space at P of the Riemannian manifold $T_{X}P_{n}$ to the tangent space $P_{n}$ :


$$SA=\exp_{G}\left(A\right)=P^{\frac{1}{2}}\exp\left(P^{-\frac{1}{2}}{AP}^{-\frac{1}{2}}\right)P^{\frac{1}{2}}\text{,}$$

$$A=\log_{P}\left(SA\right)=P^{\frac{1}{2}}\log\left(P^{-\frac{1}{2}}{\mathrm{SAP}}^{-\frac{1}{2}}\right)P^{\frac{1}{2}}\text{,}$$

**Figure 4:**
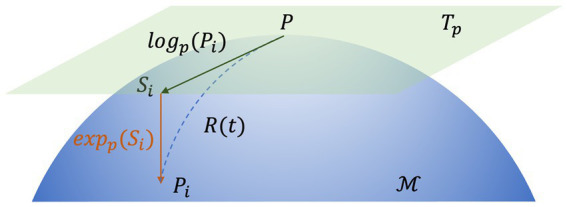
The map of the tangential space of point P. Si is the tangent vector at point P and Γ(t) is the geodesic between P and Pi.

Here, log(.) and exp(.) denote the logarithm matrix and exponential matrix, respectively. This process is illustrated in Figure 4.

By solving the minimization problem of the sum of squared Riemann distances, we obtain the geometric mean of I ≥ 1 SPD matrices in the Riemannian sense:


$$G\left(P_{1},\ldots ,P_{I}\right)=\underset{P\in P\left(n\right)}{\arg\min}\overset{I}{\underset{i=1}{\sum }}\;\delta_{R}^{2}\left({P,P}_{i}\right)$$

For a manifold $P_{n}$ with non-positive sectional curvature, there exists a unique local minimum; however, there is no closed-form expression to compute the mean. Therefore, iterative optimization algorithms must be applied to estimate the Riemannian mean (Algorithm 1).

**Table:** 

Algorithm 1: Geometric mean of SPD matrix.
Input: Ω , A collection of I SPD matrices $P_{i}\in P_{n}$ and ϵ>0
Output: $P_{\Omega }$ , Estimated mean of $P_{n}$
1:**Initialization,** $P_{\Omega }^{\left(1\right)}=\frac{1}{m}\overset{m}{\underset{i=1}{\sum }}P_{i}$
2:**repeat**
3: $S=\frac{1}{m}\overset{m}{\underset{i=1}{\sum }}\;\log_{P_{\Omega }^{\left(t\right)}}\left(P_{i}\right)$ , Arithmetic mean of tangent space
4: $P_{\Omega }^{\left(t+1\right)}={Exp}_{P_{\Omega }^{\left(t\right)}}\left(S\right)$
5:**until** $∥S{∥}_{F}<\epsilon$

For each new MI EEG trial, the Riemannian distance between the unknown SPD matrix and each within-class Riemannian mean is computed separately. Then, the minimum distance criterion is used to determine which class the new trial belongs to. However, SPD matrices cannot be directly fed into vector-based classifiers, which are efficient and popular classifiers. To address this, the concept of tangent space within the Riemannian framework allows for vectorization of the SPD matrices. By utilizing the tangent space $P_{G}=G\left(P_{i},i=1\ldots I\right)$ , which is based on the geometric mean of the entire dataset, each $P_{i}$ of SCM is mapped to this tangent space to generate a set of n(n + 1)/2-dimensional tangent vectors $s_{i}$ :


$$s_{i}=\mathrm{upper}\left(P_{G}^{-\frac{1}{2}}\log_{P_{G}}\left(P_{i}\right)P_{G}^{-\frac{1}{2}}\right)$$

Applying the $\mathrm{upper}\left(\text{.}\right)$ operator to preserve the Riemannian distance equal to the Euclidean norm, these tangent vectors form a set of features in the Riemannian space. The specific steps for Riemannian space feature extraction are presented in Algorithm 2. In this study, the dimensionality of the Riemannian tangent space (TS) features extracted from each MI trial is 2080, *n* = 64.

**Table:** 

Algorithm 2: Tangential space mapping.
Input: Ω , A collection of *I* SPD matrices $P_{i}$
Output: Θ , A collection of *I* vectors $s_{i}$
1:Compute the Riemannian geometric mean of the entire set, $P_{G}=G\left(P_{i},i=1\ldots I\right)$
2:**for** i = 1 to *I***do**
3: $s_{i}=\mathrm{upper}\left(P_{G}^{-\frac{1}{2}}\log_{P_{G}}\left(P_{i}\right)P_{G}^{-\frac{1}{2}}\right)$ 4:**end for**
5:**concatenate** $s_{i}$ to Θ
6:**return** Θ

### 2.5. Classification

Support Vector Machine (SVM) is a commonly used linear classifier in BMI applications (Subasi and Gursoy, 2010). Given a set of labeled feature vectors, this classification technique aims to separate the data by finding a hyperplane that maximizes the margin, which is the distance between the hyperplane and the nearest points from each class. We consider the margin based on Riemannian geometric features as a hard margin, indicating linear separability. Therefore, an SVM classifier based on a linear kernel function is adopted, along with a penalty term of 0.1 to achieve a model that approximates the optimal classification plane and prevents overfitting. Assuming the linear equation that defines the partitioning hyperplane is $w^{T}x+b=0$ , where $w=\left(w_{1},w_{2},\ldots ,w_{n}\right)$ represents the normal vector determining the orientation of the hyperplane, and b is the displacement term determining the distance between the hyperplane and the origin.

Clearly, the partitioning hyperplane is determined by the normal vector w and the displacement b. The distance from a sample to the hyperplane can be defined as:


$$r=\frac{\left|w^{T}x_{i}+b\right|}{\left|\left|w\right|\right|}=\frac{y_{i}\left(w^{T}x_{i}+b\right)}{\left|\left|w\right|\right|}$$

The distance from the sample set to the hyperplane is given by:


$$\rho =\min_{\left(x_{i},y_{i}\right)\in S}\frac{y_{i}\left(w^{T}x_{i}+b\right)}{\left|\left|w\right|\right|}=\frac{a}{\left|\left|w\right|\right|}$$

Maximizing the margin is our optimization objective, and its calculation formula is as follows:


$$\max_{w,b}\frac{a}{\left|\left|w\right|\right|}s.t.y_{i}\left(w^{T}x_{i}+b\right)⩾a,\forall i$$

Let $\hat{w}=\frac{w}{a},\hat{b}=\frac{b}{a}$ , the optimization objective is transformed to:


$$\max_{w,b}\frac{1}{\left|\left|\hat{w\;}\right|\right|}s.t.y_{i}\left({\hat{w\;}}^{T}x_{i}+\hat{b\;}\right)⩾1,\forall i$$

The transformed objective function does not affect the predictive performance of the model:


$$h\left(x\right)=sgn\left(w^{T}x+b\right)=sgn\left(a{\hat{w\;}}^{T}x+a\hat{b}\right)≅\hat{h}\left(x\right)\left(a>0\right)$$

For the convenience of subsequent differentiation, we improve the equation as follows:


$$\max_{w,b}\frac{2}{\left|\left|\hat{w\;}\right|\right|}s.t.y_{i}\left({\hat{w\;}}^{T}x_{i}+\hat{b\;}\right)⩾1,\forall i$$

The one-vs-one classification strategy is utilized, where SVMs are designed between any two classes of samples. After distinguishing between all possible pairs, a voting mechanism is employed to tally the number of votes for each class. Hence, for the k classes of samples, K(K-1)/2 SVMs need to be designed. When classifying an unknown sample, the class with the highest number of votes is assigned to the unknown sample.

## 3. Results

### 3.1. Traceability results

We attempted to map ROIs by finding the dipoles that best represent the MI task. In the Figure 4, the dipole power for each MI task is superimposed separately on the rendered brain to highlight the distribution in terms of the anatomical model. For all fifteen participants, there was clearly one component most relevant to the motor task; subsequent ROIs localized to the left sensorimotor cortex. We also noticed that some dipoles are important for multiple tasks.

**Figure 5:**
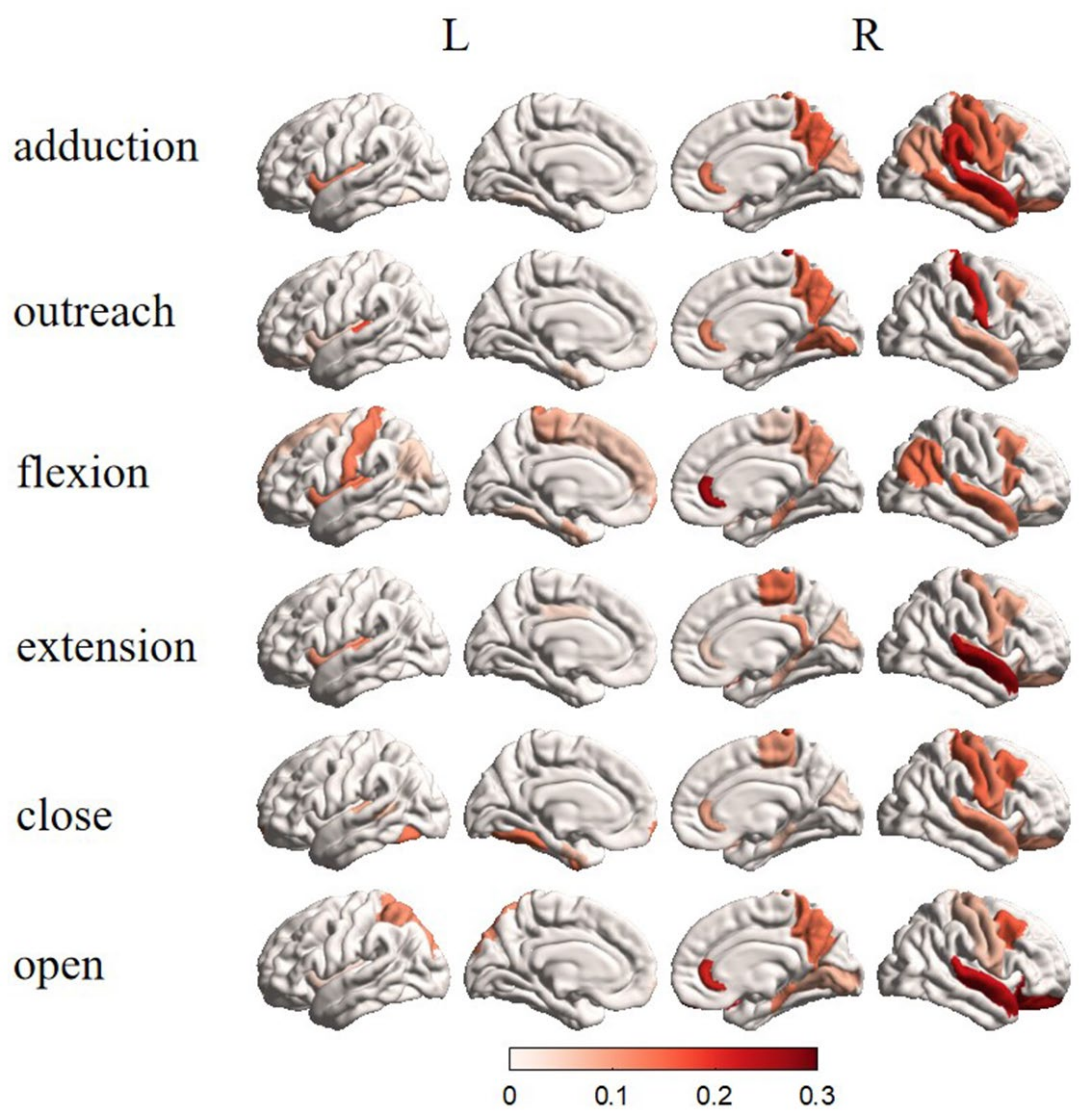
Brain topographic maps in different states after traceability.

From Figure 5, the results of source tracing were based on the template for Desikan–Killiany functional parcellation for cortical mapping (Alexander et al., 2019). Clear lateralization was evident in the right hemisphere for the overall activation across brain regions. Specifically, in the Adduction task, the activation was mainly focused on the central anterior and posterior gyri, superior temporal gyrus, superior parietal gyrus, and upper portion of the middle frontal gyrus. The Outreach task mainly activates the central posterior gyrus, superior temporal gyrus, and upper portion of the middle frontal gyrus in the right hemisphere. In the Up task, activation was predominantly observed in the left hemisphere central posterior gyrus, right hemisphere’s superior temporal gyrus, superior parietal gyrus, and upper portion of the middle frontal gyrus. The Down task predominantly activates the central anterior gyrus, superior temporal gyrus, and upper portion of the middle frontal gyrus in the right hemisphere. The Close task mainly activates the central anterior and posterior gyri, superior temporal gyrus, and upper portion of the middle frontal gyrus in the right hemisphere. The Open task predominantly activates the central anterior and posterior gyri, superior temporal gyrus, and upper portion of the middle frontal gyrus in the right hemisphere. Overall, apart from differing activation patterns between the Up and Down tasks, there is a certain degree of similarity in the activation patterns of other tasks, with differences primarily observed in activation intensity.

**Figure 6:**
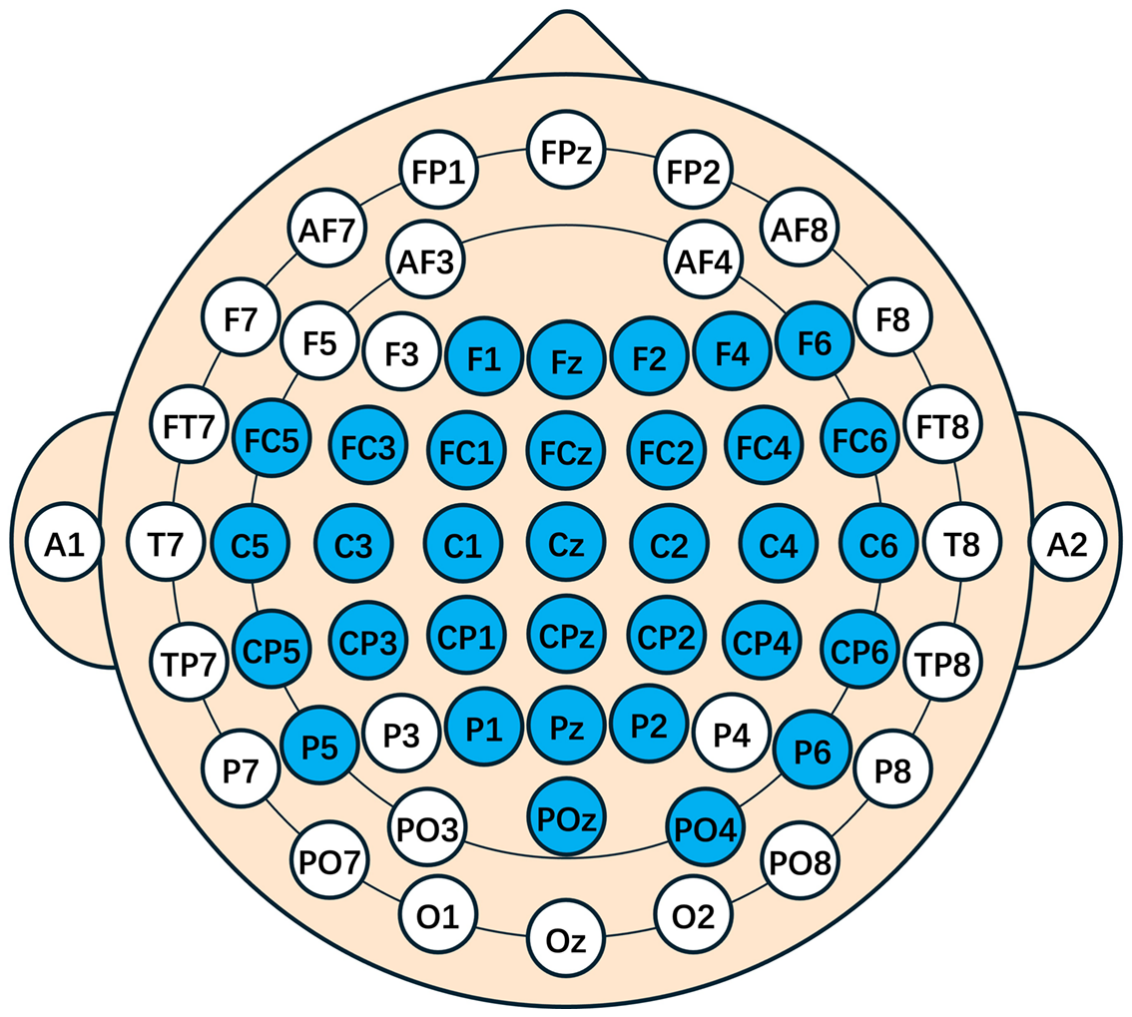
A spatial topology map after traceability. The blue indicates the electrodes that are preserved, representing the presence of source components that are highly relevant to the task in this area. The space model is the international 10–20 system.

After source localization, the mapping of the cerebral cortex to the scalp points was performed, and additional electrode positions in the motor areas were symmetrically added. The following electrodes were retained: F1, Fz, F2, F4, F6, FC5, FC3, FC1, FCz, FC2, FC4, FC6, C5, C3, C1, Cz, C2, C4, C6, CP5, CP3, CP1, CPz, CP2, CP4, CP6, P5, P1, Pz, P2, P6, POz, PO4, as shown in the spatial topology in Figure 6.

### 3.2. Classification results

Each subject’s EEG data from all trials were separately shuffled twice, and the shuffled data were then divided into training and testing sets using a 10-fold cross-validation method. This process was repeated 10 times with different random seeds for shuffling each time. The accuracy and standard deviation of all iterations were computed to obtain the experimental validation results. The experiments were conducted independently for each subject, and the training and testing sets were derived from the same subject’s dataset.

Since this is a small-sample classification study, we conducted *a priori* simulations of significance tests for different sample sizes. This was of exceptional significance to supplement the validation of our method’s superiority. As shown in the table, we obtained significance *p*-values and confidence levels for various sample sizes.

**Figure 7:**
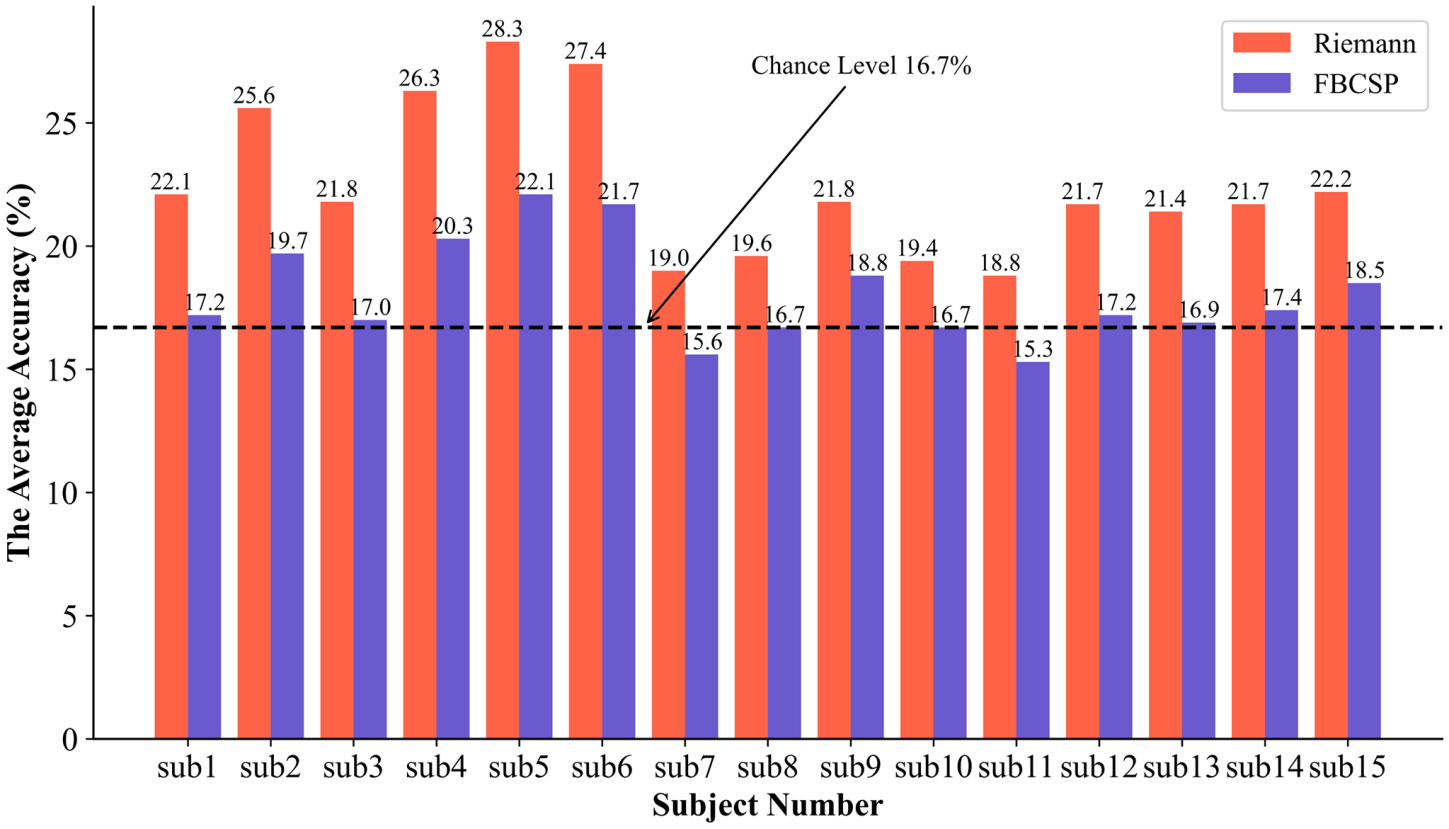
The results of the comparison experiment between Riemannian and FBCSP.

Based on the aforementioned experimental procedure and within the same source localization space, the Filter Bank Common Spatial Patterns (FBCSP) method, which is a widely used and efficient motor imagery feature extractor, was adopted for comparison with the proposed method in this study. In the FBCSP method, the EEG data in the frequency band of 8 Hz to 30 Hz were divided into 2 sub-bands with intervals of 11 Hz, and the number of CSP components was set to 4. The comparative results of these two methods are shown in Figure 7.

Our proposed method achieved an average recognition accuracy of 22.47% for the six different motor imagery tasks, while the average recognition accuracy of FBCSP was 18.07%. Comparing each subject’s results, our method exceeded the popular motor imaging algorithm by 2.7%–6.2%. In addition, corresponding to Table 1, we achieved an average classification accuracy with a significant statistical difference at the *p* < 0.01 level. Notably, there were highly significant statistical differences among participants 2, 4, 5, and 6. The optimal classification level was found to be 28.3%, and during the training process, certain data from this participant even reached an accuracy of 44.7%. However, it’s important to note that there were no statistically significant differences in classification among the other four participants.

**Table 1:** In the conditions with sample sizes ranging from 60 to 360, statistically significant classification accuracy was observed.

Sample size	*p* < 0.05	*p* < 0.01	*p* < 0.001	*p* < 0.0001
	Accuracy (%)			
60	25.00	28.33	33.33	36.67
120	22.50	25.00	27.50	30.00
180	21.11	23.33	25.56	27.78
240	20.83	22.50	24.58	26.25
300	20.33	21.67	23.67	25.00
360	20.00	21.39	23.06	24.44

This demonstrates that Riemannian geometry is better suited for handling such manifolds in the SPD space formed by the brainwave data. EEG data, to some extent, imposes limitations on Euclidean calculations. From a physiological perspective, the collected EEG signals have undergone multiple transmissions through mediums such as cerebrospinal fluid, skull, and scalp, which may cause signal distortions. Riemannian geometry defines distances differently from Euclidean space, making it more adept at handling chaotic signals. Compared to Euclidean space, the Riemannian framework can be applied on smooth and differentiable surfaces without fixing the data into a specific space. Although SPD is a prerequisite, any non-zero values can form a covariance matrix, and EEG signals happen to avoid zero values.

**Figure 8:**
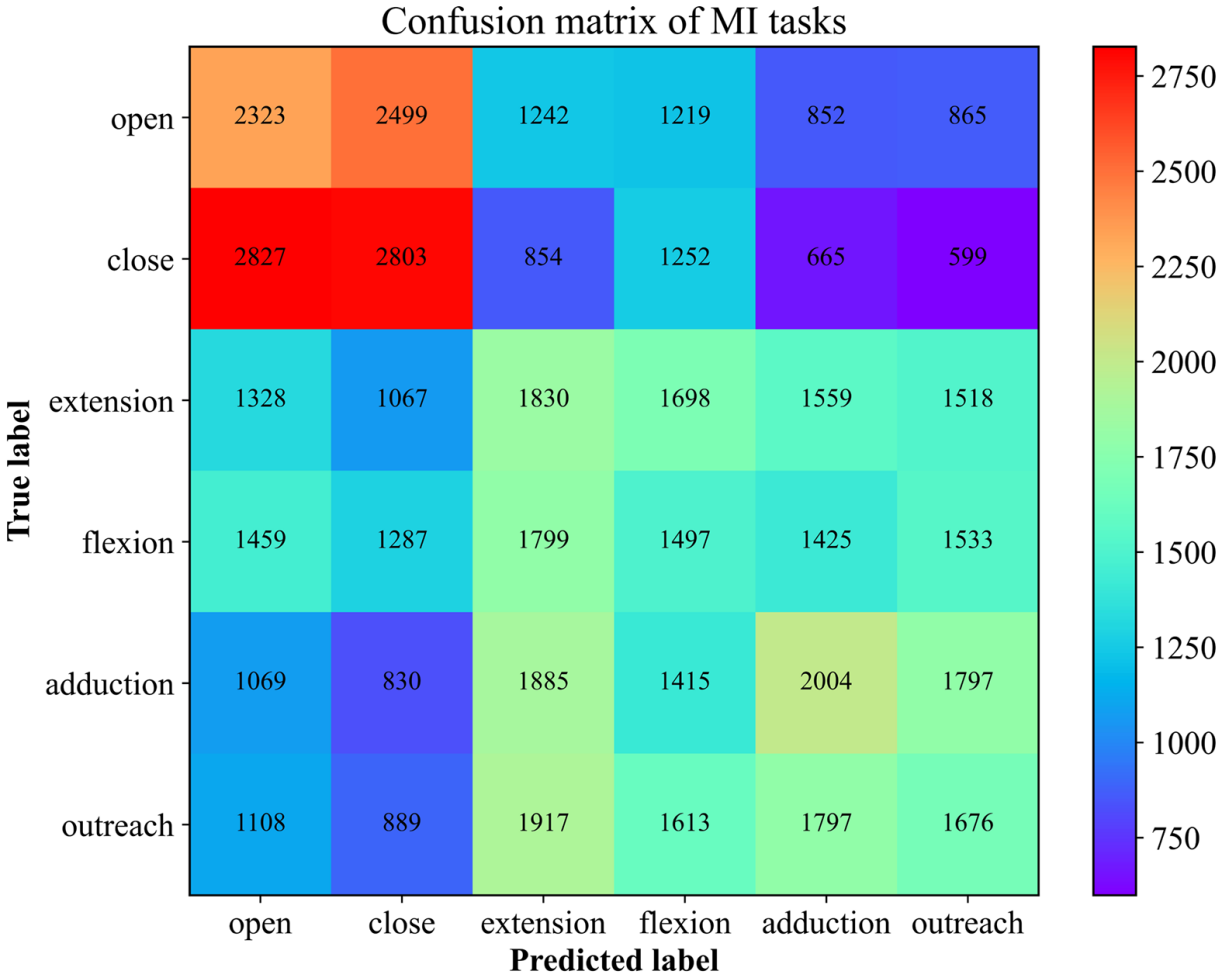
The Confusion Matrix for 6 MI Recognition. The number here corresponds to the superposition of the results predicted to be of this type in 10 times of cross-validation.

Moreover, in order to explore the prediction performance for each category, a confusion matrix (Figure 8) was used to visualize the classification results. In the confusion matrix, each row represents the actual category, and each column represents the predicted category by the classifier. The elements in the matrix indicate the number of instances classified into each predicted category by the classifier.

Combining the prediction results of all five participants, it is evident that the accuracy of predicting the “Close” motor imagery is the most significant, followed by “Open” and “Upper Arm Outreach.” However, the opening and closing of the palm are often confused. Distinguishing between the forearm’s antagonistic movements proves to be challenging, and they are often misclassified as upper arm movements to a considerable extent. This observation aligns with the differentiation of the brachial plexus nerves, as the nerves at the terminal end are more susceptible to interference from the initial end. Nonetheless, the classification algorithm is still capable of identifying these six types of motor imagery tasks to a certain extent.

### 3.3. Ablation experiment

In order to further investigate whether source localization would affect the capabilities of the feature extractor and determine in which feature extraction method the classifier can provide the optimal classification accuracy, we compared the SVM classifier with both Riemannian geometry and FBCSP. Under the same experimental conditions as the experiments with source localization, we explored the classification performance of Riemannian features and FBCSP without undergoing source localization processing.

**Figure 9:**
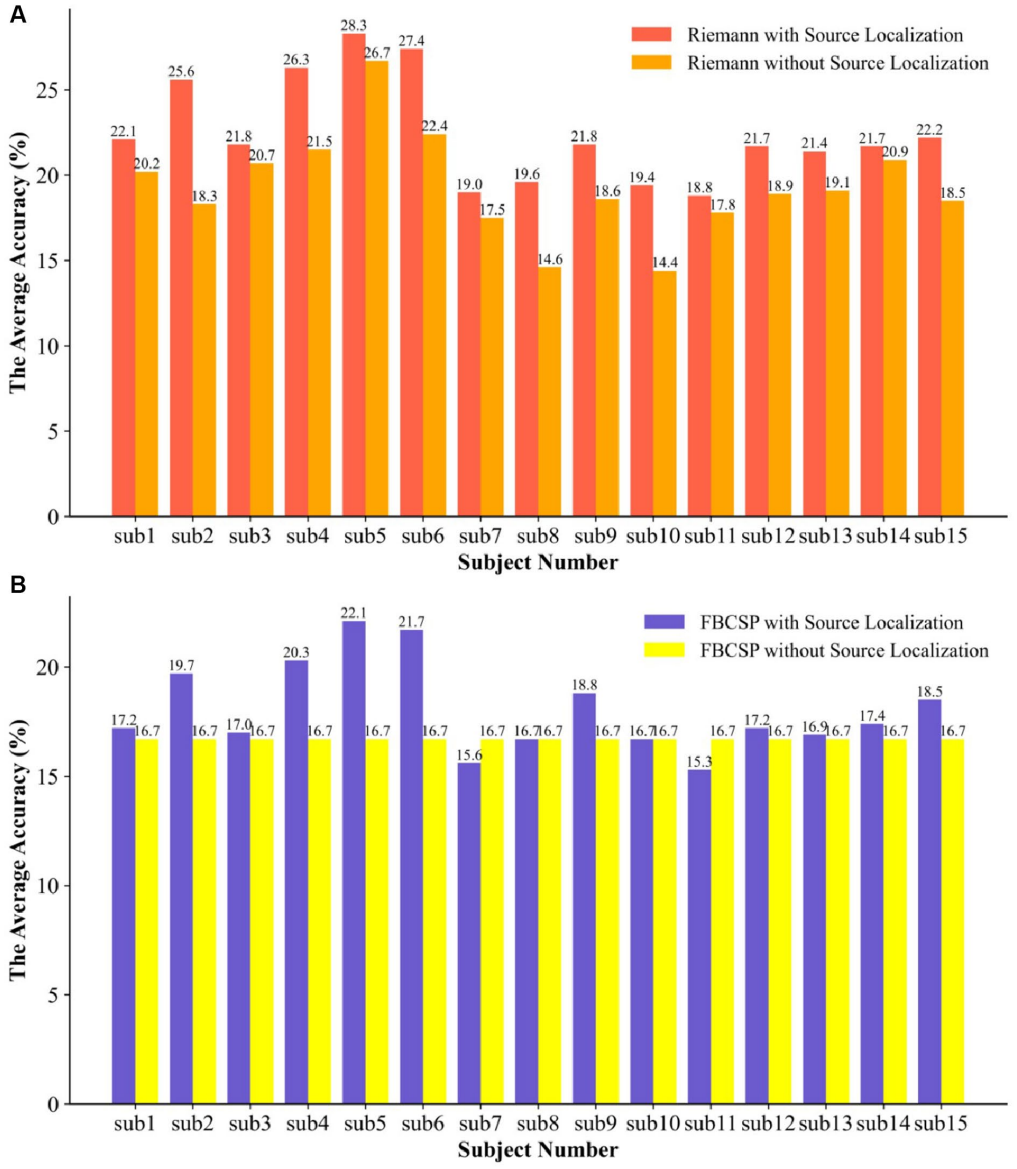
The SVM was used to compare the two types of features with and without EEG source localization. **(A)** the ablation experiment of the Riemann feature for the traceability method and **(B)** the ablation experiment of the FBCSP feature for the traceability method.

Figure 9 illustrates the classifier’s accuracy in both feature types significantly decreased without undergoing source localization. Specifically, the classifier’s performance based on Riemannian features showed an average decrease of 3.13%, and there was no statistically significant difference. In the data from participants 2, 8, and 10, there were significant declines in performance, with decreases of 7.3%, 5%, and 5%, respectively. The classifier struggled to recognize the FBCSP features, resulting in all subjects regressing to random chance levels, even pulling up the data of participants 7 and 11, leading to an overall average decrease of 1.37% in classification accuracy. Due to the two properties of affine invariance and inverse invariance of the Riemannian metric, the Riemannian method is more robust to outliers in BMIs decoders. Comparing the improvement in feature extractor performance due to source localization, the Riemannian framework slightly outperformed FBCSP, and the combination of the Riemannian framework with brain EEG source localization proved superior to the popular method. Moreover, during the model training process, both Riemannian and FBCSP features required more time for classifier training, which is evident since larger data structures lead to increased computational demands.

### 3.4. Feature robustness verification

Given the evident temporal variability in EEG, where the EEG signals can change due to daily physiological conditions, this presents a challenge for active BMIs. Therefore, it is crucial to conduct validation experiments to assess the robustness of the models.

**Figure 10:**
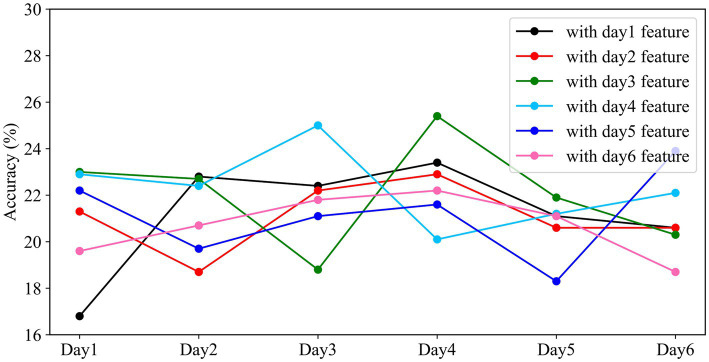
Performance of different models on cross-day data. The horizontal axis represents the experimental time, and the vertical axis represents the classification accuracy of the models with different weights on that day.

In this experiment, the data was partitioned on a daily basis, and all participants’ data for each day were combined, resulting in six datasets. The model was trained and validated on the data from the first day, and its weights were saved. The remaining days’ data were used as the testing set, and the saved weights from the first day were loaded for validation. This process was repeated for each subsequent day, where the data from the current day was used for training, and the data from the remaining days were used for testing. The results are shown in Figure 10.

From Figure 10, we observed that the overall fluctuations remained within approximately 21.39%. The standard deviations for the same model applied to different days’ data were 4.76, 1.79, 4.36, 2.29, 3.18, and 1.47, respectively. In the context of using weights trained on one day’s data to test on data from other days, an interesting pattern emerged: the accuracy of the classifier sometimes improved when tested on data from days different from the training day. Notably, when the testing interval was shorter between the training and testing days, there was greater fluctuation in classification accuracy. However, as the time interval between the training and testing days lengthened, the classification accuracy exhibited a declining trend. The weights trained on the first day showed the highest degree of fluctuation in accuracy, while those from the sixth day exhibited the least variability when applied to the classifier. This indicates that the model’s performance was more stable and consistent when using weights from the sixth day for classification.

## 4. Discussion

Our work extends the research in the field of motor imagery by investigating higher degrees of freedom in brain-machine interaction, including fist clenching, hand opening, elbow flexion, elbow extension, abduction, and adduction. However, due to the proximity of neural activation in the motor cortex during single-limb movements and the low signal-to-noise ratio in EEG signals, there are challenges in distinguishing these movements. To address these issues, the main contributions of this research involve proposing a source localization method to remove spatial noise in EEG and introducing a novel feature extraction approach based on Riemannian geometry. To evaluate the proposed method, we compared it with the widely-used FBCSP (Ang et al., 2008) method in motor imagery by applying both approaches to classify data collected from 15 subjects. The results demonstrated a significant improvement in decoding accuracy with the combination of EEG source localization and the Riemannian framework, achieving an average classification accuracy of 22.47%, and even reaching a peak accuracy of 44.7% in well-performing subjects, surpassing popular motor imagery algorithms by 4.4%. Moreover, the Riemannian framework demonstrated greater robustness in handling the outlier observed in subject 4 during decoding. This highlights the superiority of Riemannian geometry in handling the complex SPD space formed by brainwave data (Navarro-Sune et al., 2016). The distinctive capacity of Riemannian geometry lies in its ability to define distances in a manner distinct from Euclidean space, enabling it to efficiently handle chaotic signals and achieve more effective extraction of target task intention signals. Consequently, this spatial feature enhancement ultimately elevates the overall decoding accuracy of motor imagery brain-computer interface systems.

Additionally, we validated the brain EEG source localization performance of the Riemannian features and FBCSP on the same dataset. Without the spatial feature enhancement provided by brain EEG source localization, both features showed a considerable decrease in decoding performance, with Riemannian features experiencing a more significant decline. This indicates that Riemannian features themselves possess remarkably strong feature extraction capabilities and are closely related to spatial information. Regarding the source localization results, it can be inferred that these are the regions involved in upper limb movements, effectively screening out electrodes from irrelevant areas such as the frontal and occipital lobes. This work not only offers valuable insights for the study of motor imagery but also extends its potential applications to emotion analysis, psychiatric disorder diagnosis, and further advancements in brain function analysis.

In response to the issue of temporal variability in active BMIs, ensuring feature robustness becomes crucial. Therefore, in this study, the weights trained on different-day datasets were transferred and tested on datasets other than their own, the overall fluctuations in accuracy remained about 21.39%, with an overall average variance of 2.98. Interestingly, the fluctuations observed on the first and sixth days exhibited precisely opposite patterns. Riemannian features maintained a high degree of consistency with the training dataset in adjacent time periods, resulting in peaks of accuracy that even surpassed the training data. This observation underscores the importance of considering the temporal aspects of EEG data in brain-computer interface research. It suggests that there is a temporal dependency in the performance of the model, with certain weights performing better or worse depending on the time interval between training and testing. This finding could have implications for the development of BMIs and may inform strategies for improving their robustness and reliability in practical applications.

The confusion matrix visualization revealed that our method excelled in recognizing the “Close” motor imagery, followed by “Open” and “Upper Arm Outreach.” However, distinguishing between antagonistic movements of a single limb has proven to be challenging, often leading to confusion between the two types of antagonistic movements, especially forearm movement imagination. This observation aligns with the differentiation of the brachial plexus nerves, with nerves at the terminal end being more susceptible to interference from the initial end. In our future work, we aim to enhance the discrimination of this aspect. We believe that even signals generated by the same neuron may exhibit different temporal patterns. Therefore, the feature extractors in future research will place greater emphasis on capturing temporal information. Additionally, for source localization, we will employ more refined and higher-resolution spatial partitioning methods.

Overall, our research contributes valuable insights into the field of motor imagery tasks and provides a novel and efficient method for high-precision recognition. The proposed approach holds great promise for patients with motor impairment and communication impairments, enabling the development of advanced and reliable medical-assistive devices and applications.

## 5. Conclusion

This research addresses the challenges in high-precision recognition of motor intentions using EEG signals derived from motor imagery tasks. EEG signals have weak potential difference changes and are susceptible to noise and physiological influences, which limit temporal–spatial resolution. The research proposes a novel method that combines signal traceability and Riemannian geometric features to identify six motor intentions of the upper limb. The process involves separating relevant components from the original signal and tracing them to the source using standardized low-resolution brain electromagnetic tomography. The EEG data from irrelevant electrodes are screened out. Riemannian geometric features are then extracted from the covariance matrix of the reconstructed EEG signals. These features are used for pattern recognition using a support vector machine with a linear kernel function. The proposed method achieves an average accuracy of 22.47% for the six classifications on a dataset of 15 participants, the average variance of classification accuracy due to feature variation over time is 2.98%. The results demonstrate that the proposed method significantly improves intent recognition accuracy. The research shows promise for assisting patients with motor impairments and communication disabilities.

## Data availability statement

The raw data supporting the conclusions of this article will be made available by the authors, without undue reservation.

## Ethics statement

The studies involving humans were approved by the local Ethics Committee of Nanjing Tech University. The studies were conducted in accordance with the local legislation and institutional requirements. The participants provided their written informed consent to participate in this study.

## Author contributions

MZ: Conceptualization, Data curation, Investigation, Methodology, Software, Validation, Visualization, Writing – original draft. JH: Formal analysis, Investigation, Methodology, Project administration, Supervision, Writing – review & editing. SN: Formal analysis, Resources, Supervision, Validation, Writing – review & editing.
